# Immersive Virtual Reality Reminiscence Reduces Anxiety in the Oldest-Old Without Causing Serious Side Effects: A Single-Center, Pilot, and Randomized Crossover Study

**DOI:** 10.3389/fnhum.2020.598161

**Published:** 2021-01-18

**Authors:** Kazuyuki Niki, Megumi Yahara, Michiya Inagaki, Nana Takahashi, Akira Watanabe, Takeshi Okuda, Mikiko Ueda, Daisuke Iwai, Kosuke Sato, Toshinori Ito

**Affiliations:** ^1^Department of Clinical Pharmacy Research and Education, Osaka University Graduate School of Pharmaceutical Sciences, Osaka, Japan; ^2^Department of Pharmacy, Ashiya Municipal Hospital, Hyogo, Japan; ^3^Department of Systems Innovation, Osaka University Graduate School of Engineering Science, Osaka, Japan; ^4^Social Welfare Corporation Misasagikai, Osaka, Japan; ^5^Osaka Center for Cancer and Cardiovascular Disease Prevention, Osaka, Japan

**Keywords:** virtual reality, reminiscence, anxiety, satisfaction, late elderly

## Abstract

**Background:** Dementia is one the major problems of aging societies, and, novel and effective non-drug therapies are required as interventions in the oldest-old to prevent cognitive decline.

**Objective:** This study aims to examine the efficacy and safety of reminiscence using immersive virtual reality (iVR reminiscence) focusing on anxiety that often appears with cognitive decline. The secondary objective is to reveal the preference for VR image types for reminiscence: live-action (LA) or computer graphics (CG).

**Methods:** This was a pilot, open-label, and randomized crossover study which was conducted on January 2020 at a single nursing home. The subjects were randomly divided into two groups (A or B) in equal numbers, and they alternately viewed two types of VR images (LA and CG) themed on the mid- to late Showa era (A.D. 1955–1980) in Japan. In group A, the CG images were viewed first, and then the LA images were viewed (CG→ LA). In group B, the images were viewed in the opposite order (LA→ CG). Before VR viewing, subjects responded to Mini-Mental State Examination (MMSE) Japanese version and State-Trait Anxiety Inventory (STAI) Japanese version. After viewing the first and second VR, subjects responded to STAI and the numerical rating scale (NRS) for satisfaction and side effects (nausea, dizziness, headache, and tiredness).

**Results:** Ten subjects participated in this study. The values of analyses are presented in the mean (SD). The age was 87.1 years (4.2), and the MMSE was 28.5 (1.8). The total STAI score before VR viewing was 36.1 (7.2), but it significantly decreased to 26.8 (4.9) after the first VR viewing (*P* = 0.0010), and further decreased to 23.4 (2.8) after the second VR viewing (*P* < 0.001). The NRS score for satisfaction tended to be higher after viewing LA in group A (CG→ LA) (CG vs. LA; 7.0 (2.3) vs. 8.6 (1.5), *P* = 0.0993), while in group B (LA→ CG), the score after CG was slightly lower than that after LA. There were no serious side effects.

**Conclusions:** This study suggests that iVR reminiscence can reduce anxiety in the oldest-old without causing serious side effects. Furthermore, the impacts might be better with LA images.

## Introduction

Dementia is one the major problems of aging societies, and various studies are being carried out around the world to address the issue. However, no curative drug therapy for dementia has yet been established. Furthermore, in 2018, four commercially available drugs for dementia (donepezil, galantamine, rivastigmine, and memantine) were excluded from national insurance coverage in France due to their high risk of side effects, rather than their efficacy (Krolak-Salmon et al., 2018). More recently, a series of phase 3 trials of new dementia drug candidates (Salloway et al., 2014; Honig et al., 2018; Wessels et al., 2019) ended in failure, highlighting the limitations of drug therapy for dementia. On the other hand, the FINGER study (Ngandu et al., 2015) recommended that interventions should be undertaken simultaneously to prevent cognitive decline because dementia is an interrelated multifactorial disorder. In addition, Livingston et al. (2017) identified nine factors that can prevent developing dementia through self-effort, specifically, dementia onset can be delayed if lifestyle (hypertension, obesity, smoking, depression, and diabetes) is improved, physical activity is increased, and coping with social isolation is begun at the early stage of suspected cognitive decline [i.e., mild cognitive impairment (MCI)]. In addition, Barnes and Yaffe (2011) suggest that decreased physical activity and depression are more risk factors for Alzheimer's disease than lifestyle-related diseases such as diabetes. Depression and anxiety are typical behavioral and psychological symptoms of dementia (BPSD), which often occur at the stage of MCI and bring a relatively heavy care burden (Black and Almeida, 2004). Therefore, although coping with these mental symptoms is important both in the elderly with MCI and their caregivers, it is rather difficult to respond with pharmacotherapy for mental symptoms (Yury and Fisher, 2007). In addition, since it is not possible to administer prophylactically antidementia drugs at the MCI stage, some novel non-drug therapies are urgently required.

Several approaches are reported to prevent cognitive decline, such as occupational therapy (Hermans et al., 2007; Gitlin et al., 2008), exercise therapy (Laurin et al., 2001; Thomas and Hageman, 2003; Rolland et al., 2007; Santana-Sosa et al., 2008; Hauer et al., 2012), and music therapy (Ueda et al., 2013). In addition, as a psychotherapy, there is reminiscence therapy advocated by Butler (1963), and further studies using this approach have shown to reduce cognitive decline, anxiety, and depressive symptoms (Goldwasser et al., 1987; Wang, 2007; Huang et al., 2015; Lok et al., 2019). In this context, there is a growing interest in digital therapeutics (DTx), a new non-drug approach that utilizes digital technologies such as the Internet of Things, artificial intelligence (AI), and virtual reality (VR). Because DTx is characterized by its extremely high affinity for telemedicine, the demand is skyrocketing globally at the moment with the coronavirus disease 2019 (COVID-19) raging all over the world (Guan et al., 2020; Li et al., 2020; Zhu et al., 2020). DTx is expected to prevent the spread of emerging and re-emerging infections without compromising the quality of healthcare (Humphreys et al., 2020; Ohannessian et al., 2020; Rockwell and Gilroy, 2020; Wang et al., 2020).

Recently, an approach using DTx was also considered for reminiscence; however, there are still few reports. For example, Subramaniam and Woods (2016) visualized memories heard from six dementia patients and compared the effects of life story videos with those of traditional album-style life story books, suggesting that life story videos had the more potential to improve patient quality of life. In addition, Moon and Park (2020) conducted twice-weekly reminiscence sessions with 25 dementia patients for 4 weeks, using tablets PC with an app installed that allowed the subjects to select and play back a collection of favorite images of their memories *via* the Internet. The comparison the results with those of 24 participants in the conventional reminiscence group without digital devices shows that depression were significantly reduced in the group that used the digital devices immediately after the initial reminiscence and 4 weeks later. In addition, we found for the first time that usage of immersive VR (iVR) to recall memorable places improved various physical and mental symptoms, such as pain, anxiety, and depression in terminally ill cancer patients (Niki et al., 2019). VR is a generic term for technology that works on human sensory organs to artificially create a three-dimensional (3D) environment that feels like reality. Because iVR has been commercially available since 2016 and has a very short history, there are no studies comparing the effects of 2D and 3D as a memory-recalling approach like reminiscence to the best of our knowledge. However, Schutte and Stilinovi (2017) compared the effects of iVR on empathy and engagement of “being there” with a 2D monitor. The results showed that both empathy and engagement were higher for iVR experiences than for 2D monitors, suggesting that iVR is more powerful in working with emotions. Therefore, we hypothesize that the iVR could be also more effective in a reminiscence. In this study, we first examined the efficacy and safety of iVR reminiscence as a pilot study, focusing on anxiety as one of the psychiatric symptoms that often appears with cognitive decline.

## Materials and Methods

### Subjects

The inclusion criterion involves those 75 years of age or older who were using day services at a nursing home as of January 2020. The exclusion criteria were set as follows: (1) poor recognition of VR images and (2) cognitive function was too low to answer the questionnaire. Poor recognition of VR images defined as “when the subjects complain of not being able to see the image clearly and the difficulty persists even after adjusting the mounting position of the VR headset.” The too low cognitive function to answer the questionnaire defined as “when there was no coherent conversation between the questioners and subjects and the subjects were unable to answer the questions on the Likert scale of the State-Trait Anxiety Inventory (STAI) Japanese version (Iwata et al., 1998a,b) and the numerical rating scale (NRS).” Staff of the nursing home explained the study to the subjects in written form, and informed consent was obtained in writing.

### Preparation of VR Images

Two types of VR images were prepared: live-action (LA) images and computer graphics (CG) images themed on the mid- to late Showa era (A.D. 1955–1980) in Japan. The LA images were shot using a 360° camera (Insta360 Pro 2X, Arashi Vision Inc., Shenzhen, China) at *Itsuka Kita Michi* in *Miroku no Sato* (https://www.mirokunosato.com/itsuka), a theme park in Fukuyama City, Hiroshima Prefecture. *Itsuka Kita Michi* is a facility that precisely recreates elements such as arcade, elementary schools, post-offices, shopping streets, and fields of the Showa era 30's (A.D. 1955–1965) in Japan, and we took many photos to document every scene in this facility. In addition, we purchased the “Showa 80's (A.D. 1970–1980) Japanese town model set vol. 3” (FUNSET; https://assetstore.unity.com/packages/3d/environments/urban/shouwa-80-s-japanese-town-model-set-vol-3-mall-127437) from Unity asset store and edited the CG images using Unity (Unity Technologies, San Francisco, USA), a game engine that creates VR content. Six situations were set up as familiar scenes to those aged 75 and over: arcades, cafeterias, sunken hearths, dagashi shops (old Japanese candy stores), downtown, and fields.

### Operation of VR Images

**Figure 1:**
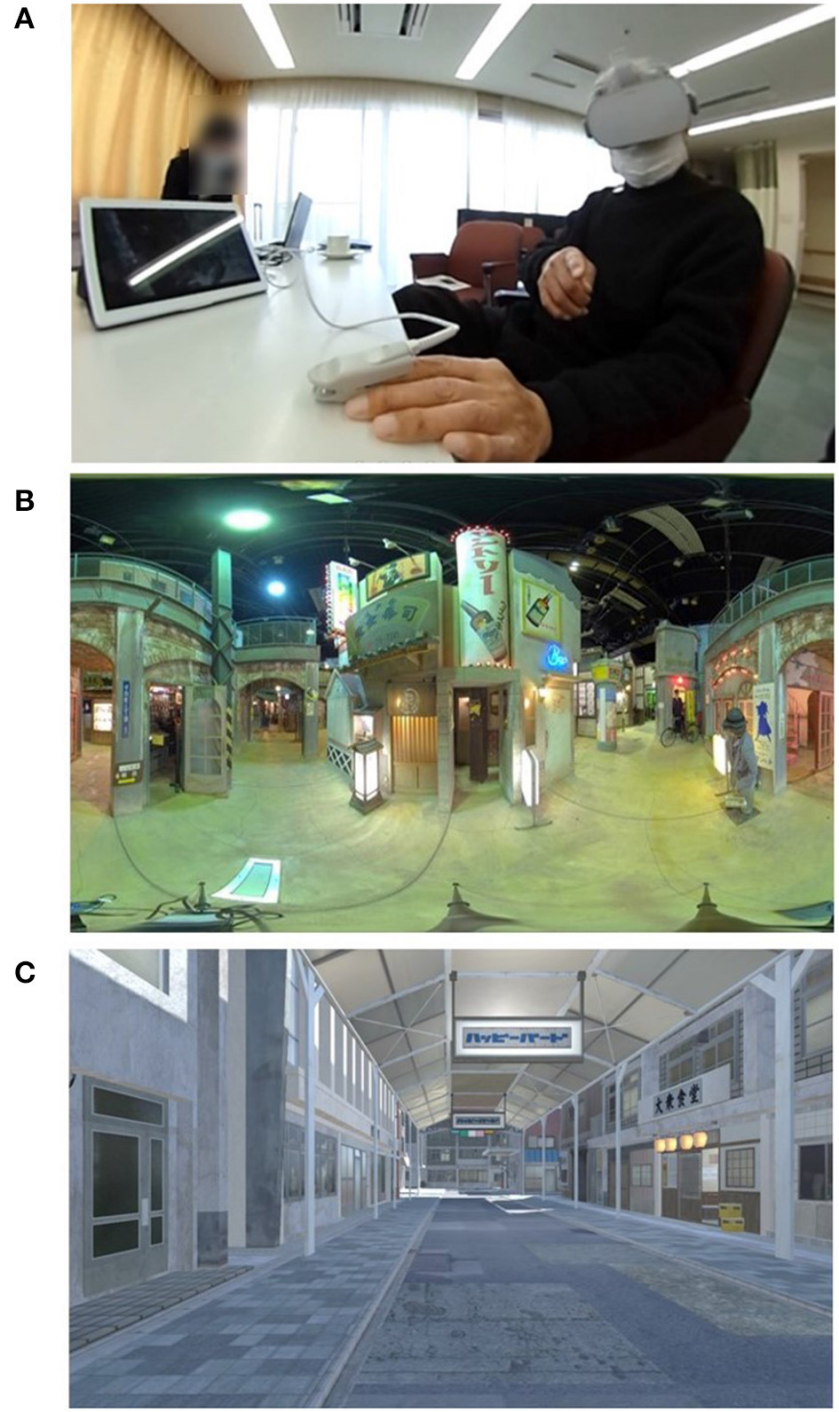
A subject during immersive virtual reality reminiscence (iVR reminiscence) **(A)** and examples of live-action (LA) and computer graphics (CG) images **(B,C)**. Two types of VR images were prepared: live-action (LA) images and computer graphics (CG) images themed on the mid to late Showa era (A.D. 1955–1980) in Japan. Oculus Go (Facebook Technologies, California, USA) was used as the VR headset. The LA images were a slide show of 54 photographs shown at 15-s intervals. The researcher pressed a button to start the slideshow, and the subjects simply had to wear a headset. For the CG images, the researcher selected the scenes based on the wishes of the subjects. The researcher performed movement operations in VR space, and the subject only had to wear the headset. By mirroring the images from the VR headset to a tablet PC, the images that the subjects were watching inside the headset could be shared. While viewing VR, there were no restrictions on conversation among the researchers and the nursing home staff, so we had natural conversations with the subjects in response to what they said.

A subject during iVR reminiscence and examples of LA and CG images are shown in Figure 1. Oculus Go (Facebook Technologies, California, USA) was used as the VR headset. The LA images were a slide show of 54 photographs, shown at 15-s intervals. The subjects simply had to wear the headset and watch. For the CG images, the researcher selected the scenes according to the subjects' wishes. The images in the VR headset were mirrored on the tablet PC. The researcher performed movement operations in VR space instead of subjects by using a controller while watching the mirrored images on the tablet PC, therefore, the subjects only had to wear the headset.

### Study Design, Implementation, and Evaluation of the iVR Reminiscence

This is a single-center, pilot, open-label, randomized crossover study. The subjects were randomly divided into two groups in equal numbers, and alternately viewed two types of iVR images (LA and CG). In group A, the CG images were viewed first, and then the LA images were viewed. In group B, the images were viewed in the opposite order. The randomization was conducted by the permuted block method. The block size was set to four subjects per block, and the allocation of each block was predetermined (AABB, BAAB, and ABAB). Before the first viewing, subjects were assessed on their current cognitive function and anxiety by the Mini-Mental State Examination (MMSE) Japanese version (Sugishita et al., 2016) and the STAI Japanese version (Iwata et al., 1998a,b). The STAI is a globally used tool for measuring adult emotions and consists of 20 questions that assess how the respondents are feeling right now. For each question of STAI, subjects responded on a 4-point Likert scale (1 = almost never, 2 = occasionally, 3 = most of the time, 4 = almost always). The total score for STAI ranges from 20 to 80, with higher scores indicating stronger anxiety. Then, subjects wore a VR headset and viewed the first VR images for 10 min. After the first viewing, subjects responded to STAI and the NRS for satisfaction and side effects (nausea, dizziness, headache, and tiredness). NRS is a tool that evaluates the degree of emotion or symptoms on a scale of 0 to 10. In this study, satisfaction was set to “0 = not at all to 10 = quite satisfied,” and side effects were set to “0 = not at all to 10 = most severe.” Then, after a 10-minbreak, the subjects viewed the second VR images for 10 min, and they received the same evaluations as the first time and answered the question, “Which images were better, the LA or the CG images?” While viewing VR, there were no restrictions on conversation among the researchers, nursing home staff, and subjects. To ensure that subjects were not nervous about their first experience of viewing iVR, the familiar nursing home staff were present throughout the experiment. In addition, if the subjects showed any unusual behavior such as excitement within 1 week of the study date, the nursing home staff would record the date, time, and condition and would contact the principal researcher.

### Primary and Secondary Endpoints

The primary endpoint was the change in total STAI scores after the second VR viewing from before viewing. The secondary endpoints were the safety of the iVR reminiscence and the preference for LA or CG images.

### Statistical Analyses

Data were collected through February 2020 and analyzed from March to April 2020. In the results, the values of analyses are presented in the mean and standard deviation (SD). Comparisons of STAI scores before and after the first VR viewing, and after second viewing were performed by a Dunnett's test with the STAI scores before the first VR viewing as control groups. A Student's *t*-test was performed to compare subjects' backgrounds and the amount of change in STAI scores between two groups. A paired *t*-test was performed for changes in satisfaction and side effects. BellCurve for Excel (Social Survey Research Information Co., Ltd., Tokyo, Japan) was used for statistical analysis, with two-tailed *P* < 0.05 as statistically significant.

### Ethical Considerations

The study was approved by the Research Ethics Review Committee of the Osaka Center for Cancer and Cardiovascular Disease Prevention (approval number; R1-RINRI-9) and was registered with the University Hospital Medical Information Network Clinical Trials Registry (UMIN000039762).

## Results

Twelve individuals were enrolled in the study, one was excluded due to poor recognition of VR images, and one was excluded from the analysis because she was unable to answer the questionnaire. The backgrounds of the 10 subjects who completed the study are shown in Table 1. The mean age was 87.1 years (4.2), the mean MMSE was 28.5 (1.8), and the lowest MMSE score was 24. Group B was significantly older than group A (*P* = 0.0299); however, there were no significant differences in other variables.

**Table 1:** Subjects' backgrounds.

**Variables**	**All subjects (*n* = 10)**	**Group A (*n* = 5)**	**Group B (*n* = 5)**	***P*-value (groups A vs. B)**
Age, years, mean [*SD* (range)]	87.1 [4.2 (82, 93)]	84.4 [2.3 (82, 88)]	89.8 [4.0 (83, 93)]	0.0299
Male [*n* (%)]	4 (40)	1 (20)	3 (60)	0.5238
Level of care needed [*n* (%)]				N/A
Requiring support 1	2 (20)	1 (20)	1 (20)	
Requiring support 2	4 (40)	2 (40)	2 (40)	
Requiring long-term care 1	2 (20)	1 (20)	1 (20)	
Requiring long-term care 2	2 (20)	1 (20)	1 (20)	
Requiring long-term care 3	0 (0)	0 (0)	0 (0)	
Requiring long-term care 4	0 (0)	0 (0)	0 (0)	
Requiring long-term care 5	0 (0)	0 (0)	0 (0)	
MMSE-J, mean [*SD* (range)]	28.5 [1.8 (24, 30)]	28.8 [0.8 (28, 30)]	28.2 [2.5 (24, 30)]	0.6233

*Student's t-test*.

*SD, standard deviation; N/A, not available; MMSE-J, Mini-Mental State Examination Japanese version*.

**Figure 2:**
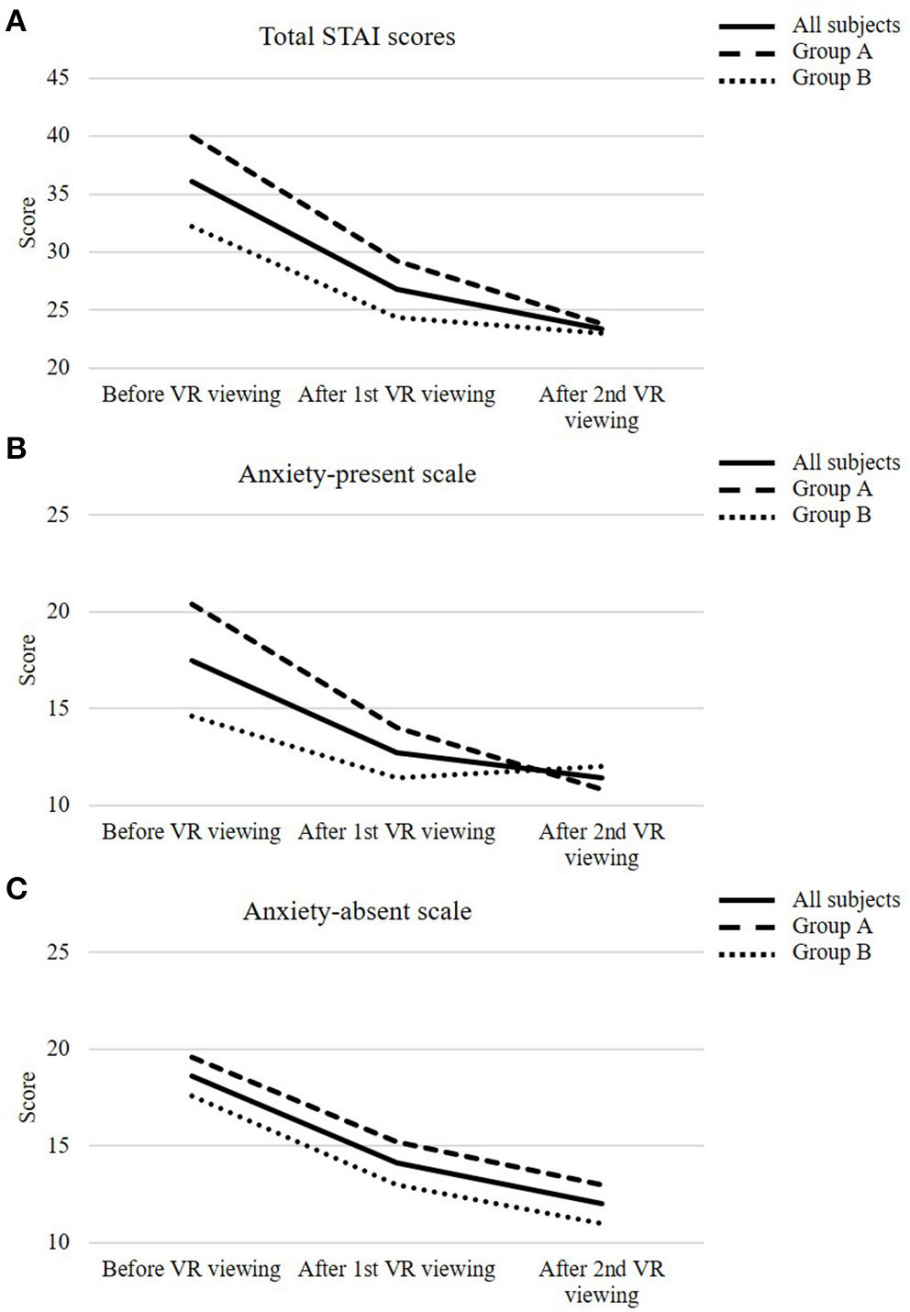
The change in State-Trait Anxiety Inventory (STAI) scores before virtual reality (VR) viewing, after the first and second VR viewing in all subjects and each group. The change in total STAI scores **(A)**, anxiety-present scale **(B)**, and anxiety-absent scale **(C)**. All subjects are represented by solid lines, and groups A and B are represented by thick and thin dotted lines, respectively. Subjects were assessed on their anxiety by the STAI Japanese version before VR viewing. Then, subjects wore a VR headset and viewed the first VR images for 10 min. After the first VR viewing, subjects responded to STAI and the numerical rating scales for satisfaction and side effects (nausea, dizziness, headache, and tiredness). Then, after a 10-min break, the subjects viewed the VR images for 10 min, which were different from the first time, and after the second VR viewing, they received the same evaluations as the first time.

**Figure 3:**
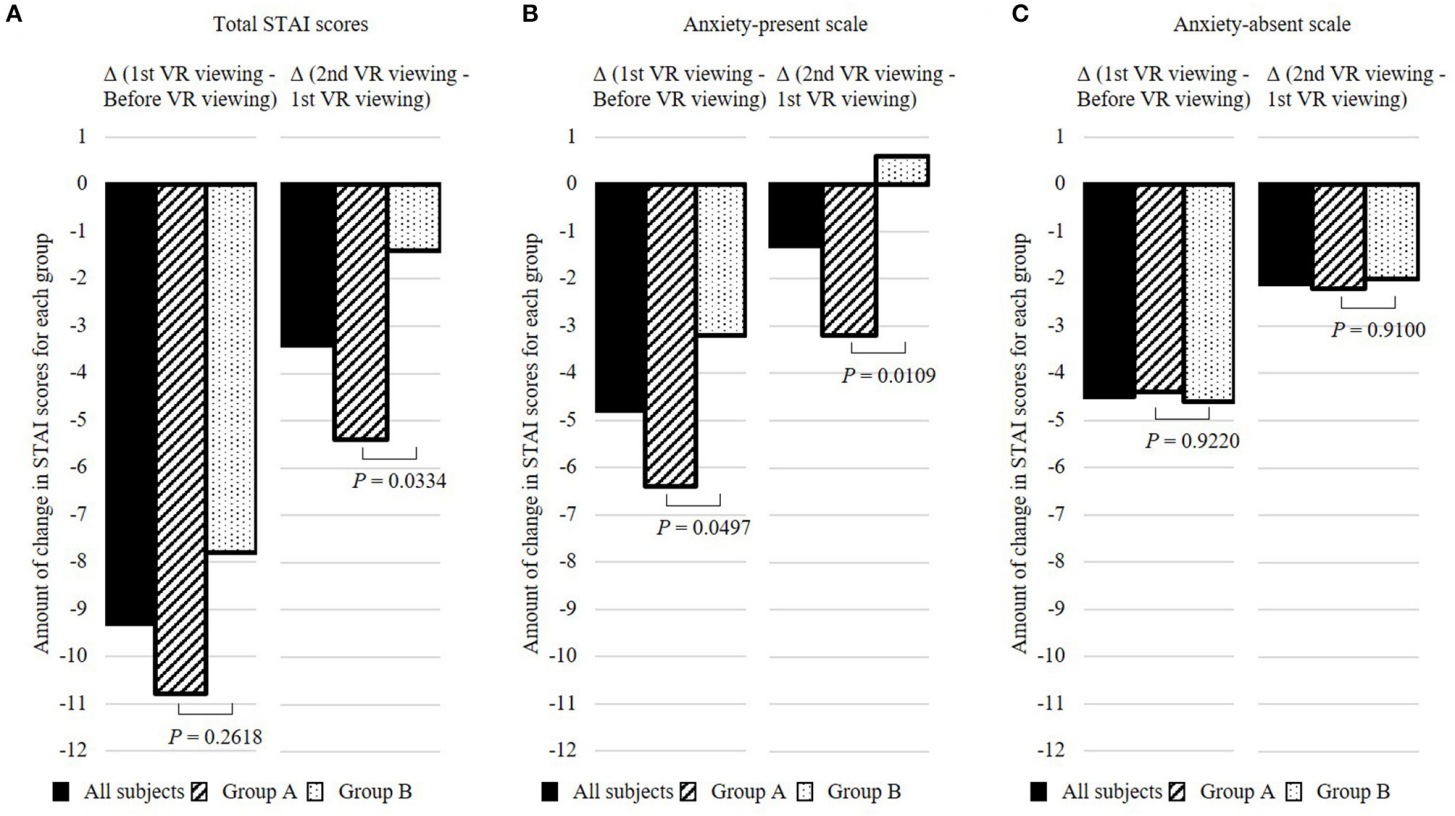
The amount of change in State-Trait Anxiety Inventory (STAI) scores in total STAI scores **(A)**, anxiety-present scale **(B)**, and anxiety-absent scale **(C)**. All subjects are represented by black squares, and groups A and B are represented by slanted and dotted squares, respectively. Subjects were assessed on their anxiety by the State-Trait Anxiety Inventory (STAI) Japanese version before and after the first VR viewing, and after second VR viewing. The amount of change in STAI scores from before VR viewing to after the first VR viewing (before VR viewing—after the first VR viewing) and the amount of change in STAI scores from after the first VR viewing to after the second VR viewing (after the first VR viewing—after the second VR viewing) were calculated.

Figure 2 shows the change in STAI scores with VR viewing, and the amount of change in STAI scores is shown in Figure 3. Regarding the change in total STAI scores on the primary endpoint (Figure 2A), the mean score was 36.1 (7.2) before viewing, but it decreased to 26.8 (4.9) after the first viewing (*P* = 0.0010), and further decreased to 23.4 (2.8) after the second viewing (*P* < 0.001).

Comparing the amount of changes in total STAI scores between the groups (Figure 3A), after the first viewing, there was a 10.8-point (3.7) decrease in group A (CG→ LA) and a 7.8-point (2.0) decrease in group B (LA→ CG) compared with before viewing. After the second viewing, there was a further decrease of 5.4 points (3.3) in group A (CG→ LA) and 1.4 points (1.7) in group B (LA→ CG), and the amount of change was significantly larger in group A than that in group B (*P* = 0.0334).

Since the STAI consists of the anxiety-present scale (P-scale), which consists of negative questions to detect the presence of anxiety, and the anxiety-absent scale (A-scale), which consists of positive questions to detect the absence of anxiety, we conducted analyses categorized by P-scale and A-scale (Figures 2B,C and 3B,C). Regarding the change in P-scales between before and after the first viewing, there was a significant decrease from 20.4 (1.9) to 14.0 (1.9) in group A (CG→ LA) (*P* < 0.001), whereas in group B (LA→ CG), the decrease was from 14.6 (4.0) to 11.4 (2.6), although not significant (*P* = 0.2481). Regarding the change in the A-scale between before and after the first viewing, there was an insignificant decrease from 19.6 (5.7) to 15.2 (3.8) in group A (CG→ LA) (*P* = 0.2049) but a significant decrease from 17.6 (1.5) to 13.0 (3.0) in group B (LA→ CG) (*P* = 0.0089). The total STAI scores, A-scale, P-scale, and the results of Dunnett's test for the all subjects, groups A and B, respectively, are shown in Supplementary Table 1.

The results of the evaluation of satisfaction and side effects of VR viewing are shown in Table 2. The NRS score for satisfaction tended to be higher after the second viewing in group A (CG→ LA) than the first viewing [first (CG) vs. second (LA); 7.0 (2.3) vs. 8.6 (1.5), *P* = 0.0993], while in group B (LA→ CG), the NRS score after the second viewing was slightly lower than the first viewing [first (LA) vs. second (CG); 8.6 (2.2) vs. 8.2 (1.9), *P* = 0.1778]. No dizziness caused by VR viewing was observed. For nausea, one subject in group A (CG→ LA) reported NRS = 1 after viewing CG, but the score became 0 after viewing LA. For tiredness, in group A (CG→ LA), one subject reported NRS = 3 after viewing CG, but the score became 0 after viewing LA, while in group B (LA→ CG), one subject reported NRS = 1 after viewing LA, and the score did not change after viewing CG. Regarding headache, one subject in group A (CG→ LA) reported NRS = 1 after viewing CG and there was no change after viewing LA. None of the subjects reported any unusual behavior such as excitement within one week of the study date. Besides, for the question; “Which images were better, the LA or the CG images?,” six subjects answered that they preferred LA images.

**Table 2:** Evaluation of satisfaction and side effects after virtual reality (VR) viewing.

	**Group A (*****n*** **=** **5)**			**Group B (*****n*** **=** **5)**		
	**After 1st VR viewing (CG)**	**After 2nd VR viewing (LA)**	***P*-value**	**After 1st VR viewing (LA)**	**After 2nd VR viewing (CG)**	***P*-value**
Satisfaction [mean (*SD* (range))]	7.0 [2.3 (5, 10)]	8.6 [1.5 (7, 10)]	0.0993	8.6 [2.2 (5, 10)]	8.2 [1.9 (5, 10)]	0.1778
Nausea [mean (*SD* (range))]	0.2 [0.4 (0, 1)]	0.0 [0.0 (0, 0)]	0.3739	0.0 [0.0 (0, 0)]	0.0 [0.0 (0, 0)]	N/A
Dizziness [mean (*SD* (range))]	0.0 [0.0 (0, 0)]	0.0 [0.0 (0, 0)]	N/A	0.0 [0.0 (0, 0)]	0.0 [0.0 (0, 0)]	N/A
Headache [mean (*SD* (range))]	0.2 [0.4 (0, 1)]	0.2 [0.4 (0, 1)]	N/A	0.0 [0.0 (0, 0)]	0.0 [0.0 (0, 0)]	N/A
Tiredness [mean (*SD* (range))]	0.8 [1.3 (0, 3)]	0.0 [0.0 (0, 0)]	0.2420	0.2 [0.4 (0, 1)]	0.2 [0.4 (0, 1)]	N/A

*Paired t-test*.

*VR, virtual reality; CG, computer graphics; LA, live action; SD, standard deviation; N/A, not available*.

## Discussion

In this study, we explored efficacy and safety of iVR reminiscence for the oldest-old, focusing on anxiety as one of psychiatric symptoms that often appears with cognitive decline. Moreover, we also examined the preference for VR image types for reminiscence. We found that iVR reminiscence could transiently reduce anxiety without causing serious side effects, and the impact might be better with LA images than CG. This is the first medical report that examined not only the efficacy and safety of iVR reminiscence but also the preference for image types in the oldest-old.

Only one subject had a suspected MCI [MMSE ≤27 (Kaufer et al., 2008; Saxton et al., 2009)], so it was estimated that most subjects' cognitive functioning was relatively preserved.

In this study, the total STAI score decreased by a mean of 12.7 points after the second viewing. In terms of the minimum amount of change that can be interpreted as to how much the score changes would be clinically meaningful to the patient, i.e., the minimal clinically important difference (MCID), Corsaletti et al. (2014) reported that the MCID for STAI was 10 points. Belland et al. (2017) reported that for elderly people with a mean (*SD*) age of 73 years (6), the total STAI scores decreased by a mean (*SD*) of 10.00 points (12.29) after music therapy. Besides, Chirico et al. (2020) investigated three groups of breast cancer patients: a group viewing nature images in iVR, a music therapy group, and a nonintervention group, and reported that the total STAI score before and after each intervention was 6.85 and 3.33 points down in the VR group and music therapy group, respectively, indicating that iVR intervention was more effective than music therapy in reducing anxiety. Of course, it is necessary to directly compare iVR reminiscence with conventional one, but iVR reminiscence might be expected to have an anxiety-reducing effect comparable with conventional non-drug therapies.

Meanwhile, it is essential to share the images that the subjects are viewing together at the same time because many reminiscences are performed with several people. Ferguson et al. (2020) evaluated the effect in a study with 25 dementia patients in a hospice (mean age 85 years) who viewed 360° iVR images of a sandy beach and reported the limitations of this the study, which were that the researchers could not know what the subjects were watching in a VR headset and thus could not response to subjects. We devised a way to share the images which the subjects were watching by mirroring the images on the tablet PC so that the researchers and nursing home staff could communicate smoothly with the subjects. This may also have contributed to the reduction of STAI score larger than the MCID.

Furthermore, no serious side effects were observed in this study. This result supports previous reports that iVR did not cause serious side effects even in people older than 70 years. In the report by Ferguson et al. (2020), two of the 25 patients with dementia exhibited unusual behavior during 3–5 h after viewing of iVR. In addition, our previous study with 20 terminal cancer patients (mean age 72.3 years) did not show any serious side effects associated with iVR viewing (Niki et al., 2019). Comparable studies are limited because there are few studies worldwide using iVR with subjects over 70 years old, but of the two studies above, Ferguson et al. (2020) used images of a beach and Niki et al. (2019) used 3D photos (still images) from Google earth VR, respectively. In other words, there was little movement of the images, and the risk of nausea (VR motion sickness), which is a side effect of most concern in VR, was considered to be small. Since more movement was found in CG used in this study, the nausea was of concern, but none of the subjects complained of nausea after viewing iVR.

In addition, we also take a guess whether LA or CG is more suitable for use in iVR reminiscence for future research in this pilot study. While LA has the advantage of being able to quickly, inexpensively, and easily create realistic VR contents, the disadvantage of being impossible to recreate nonexistent scenery. CG can create nonexistent scenery, but it takes a lot of time and cost to create realistic VR contents. The results of this study show that LA might have better effects than CG. This may be due to the result that LA images worked more on the positive emotions. One of the reasons for this result is that the NRS score for satisfaction tended to be higher after viewing LA in group A (CG→ LA). On the contrary, in group B (LA→ CG), the NRS score tended to decrease after viewing CG. In addition, tiredness was relieved after viewing LA in group A (CG→ LA), but not in group B (LA→ CG), and slightly more subjects (6/10) responded that they would prefer LA. For the second reason, the comparisons of STAI scores before and after the first viewing showed that the change in A-scales of STAI reflecting positive emotions significantly decreased in group B (LA→ CG), whereas that in group A (CG→ LA), was not significant. This may be due to the difference of personalities of the subjects between two groups. Namely, the mean (*SD*) of total STAI score before the first viewing in group A [40.0 (7.3)] tended to be higher than that in group B [32.2 (5.1)] (*P* = 0.086). There might be some subjects who felt nervous about their first VR experience in group A because some subjects told us, “I am feeling nervous because it is my first experience.” Meanwhile, some subjects in group B said, “I will try anything.” In this way, most subjects of group B tended to actively participate in this session. The decrease in P-scales of STAI reflecting negative emotions in group A after the first viewing may be due to the subjects' understanding what VR was, resulting in a less nervous state. Meanwhile, the subjects in group B did not show much nervousness even before viewing, so there was no significant change in the P-scales associated with first viewing. Therefore, the significant decrease in A-scale in group B (LA→ CG) could be purely due to the positive effect of iVR reminiscence. In fact, perhaps because group A (CG→ LA) had the mental capacity to enjoy the content of the iVR after the first viewing, there was a significant decrease in A-scale after the second viewing. In addition, in group B, P-scales increased after viewing CG compared with viewing LA, which was the exact opposite of the results in group A. For the third reason of the result that LA images worked more on the positive emotions may be due to the “uncanny valley phenomenon” (Mori et al., 2012) that the closer the animation is to the real world, the more uncanny it becomes. In fact, some subjects said, “It looks like a ghost town and it's creepy” while viewing the CG images. One method of reminiscence, which especially evokes pleasant memories of the past, is called mental time travel (MTT). Since MTT has been shown to cause activation of the hippocampus in the medial temporal lobe Milner et al. (1998), it is supposed to be important to show how VR images work on positive emotions.

There are some limitations to this study. First, because this study was a randomized crossover trial, there might have been a carryover effect from the first viewing and it cause time-dependent reduction of stress derived from familiarization with new iVR experience. Thus, consideration of appropriate washout time and comparison with traditional reminiscence will be necessary. Besides, the number of subjects was very small, thus, future studies in which the sample size is calculated based on the results of this study are needed. Second, we assessed only transient changes of anxiety. Third, some VR images were different from those of the subjects' own memories. Generic themes and scenes are usually used in reminiscence, so this is partly unavoidable. However, it has been shown that life review, which is a method to recall personal memories, can alleviate psychiatric symptoms in the elderly (Korte et al., 2012; Preschl et al., 2012). Therefore, whether it is possible to realize a tailor-made reminiscence by utilizing novel technologies such as AI will be a challenge for VR reminiscence. Fourth, the lack of side effects was predictable because of the static and short-time iVR expositions, thus, safety or tolerability of iVR might not be assessed truly. For example, the maximum amount of time that can be viewed without feeling nauseated remains unclear. Fifth, although there were no significant differences between the two groups, the degree of anxiety at baseline was different. We tried to alleviate some of the nervousness about meeting the researchers and viewing iVR for the first time by having familiar nursing home staff present with the subjects throughout the study, but we were unable to completely relieve the nervousness from some subjects. Thus, it is necessary to set up using the STAI score as a criterion at the time of recruitment and to divide the subjects into groups so that their backgrounds are as similar as possible. Finally, because the subjects were not limited to people with MCI, additional studies in people with MCI is needed in the future.

## Conclusions

This study suggests that iVR reminiscence may be a novel method to reduce anxiety in the oldest-old. Since this study is a pilot study and includes important theoretical and methodological questions that should be solved, for example, it is necessary to enrich the content based on LA images and to examine the efficacy and safety of continued interventions from multiple perspectives in future studies. However, as one of the new forms of healthcare in the rapidly aging modern world and during and the current era coexist with the COVID-19, it is now increasingly important to promote DTx to enable remote healthcare. The rapid accumulation of the evidence on DTx for preventing cognitive decline is desired because DTx could contribute to a global improvement in the quality of healthcare, as they have the potential to provide borderless, high-quality healthcare through the Internet.

## Data Availability Statement

The raw data supporting the conclusions of this article will be made available by the authors, without undue reservation.

## Ethics Statement

The studies involving human participants were reviewed and approved by the Research Ethics Review Committee of the Osaka Center for Cancer and Cardiovascular Disease Prevention (approval number; R1-RINRI-9). The patients/participants provided their written informed consent to participate in this study.

## Author Contributions

KN and TI: concept and design. KN, MY, MI, NT, and AW: acquisition, analysis, or interpretation of data. KN and MY: drafting of the manuscript. MU, DI, KS, and TI: critical revision of the manuscript for important intellectual content. KN: statistical analysis. TI: obtained funding and supervision. MI, AW, DI, KS, and TO: administrative, technical, or material support. All authors contributed to the article and approved the submitted version.

## Conflict of Interest

TI reported grants from Daikin Industries, Ltd. The authors declare that the research was conducted in the absence of any commercial or financial relationships that could be construed as a potential conflict of interest.
