# Supplementary material for: Immersive Virtual Reality Reminiscence Reduces Anxiety in the Oldest-Old Without Causing Serious Side Effects: A Single-Center, Pilot, and Randomized Crossover Study
Source: Front Hum Neurosci. 2021 Jan 18;14:598161. doi: 10.3389/fnhum.2020.598161 (PMC7849024; doi:10.3389/fnhum.2020.598161)
Supplement: Supplementary file 1 [file Data_Sheet_1.PDF]

Supplemental Table 1. The change in State-Trait Anxiety Inventory (STAI) scores before virtual reality (VR) viewing, after the first and second VR viewing in all subjects and each group.

		Total STAI scores			Anxiety-present scale			Anxiety-absent scale		
		Before VR viewing	After 1st VR viewing	After 2nd VR viewing	Before VR viewing	After 1st VR viewing	After 2nd VR viewing	Before VR viewing	After 1st VR viewing	After 2nd VR viewing
All subjects (n=10)	Mean (SD)	36.1 (7.2)	26.8 (4.9)	23.4 (2.8)	17.5 (4.2)	12.7 (2.5)	11.4 (2.2)	18.6 (4.1)	14.1 (3.4)	12 (2.1)
	P-value	-	0.0010	<0.001	-	0.0038	<0.001	-	0.0096	<0.001
Group A (n=5)	Mean (SD)	40 (7.3)	29.2 (4.4)	23.8 (1.9)	20.4 (1.9)	14 (1.9)	10.8 (0.8)	19.6 (5.7)	15.2 (3.8)	13 (2.2)
	P-value	-	0.0103	<0.001	-	<0.001	<0.001	-	0.2049	0.0496

Group B	Mean (SD)	32.2 (5.1)	24.4 (4.4)	23 (3.7)	14.6 (4.0)	11.4 (2.6)	12 (3.1)	17.6 (1.5)	13 (3.0)	11 (1.4)
(n=5)	P-value	-	0.0303	0.0122	-	0.2481	0.3763	-	0.0089	<0.001

Dunnett’s test, vs before VR viewing, abbreviation: VR, virtual reality; SD, standard deviation
